# Structured Palliative Care Training Enhances Nursing Competence: Evidence from Breast Cancer Care

**DOI:** 10.1089/pmr.2024.0061

**Published:** 2025-04-29

**Authors:** Yanping Niu, Ling Li, Qiaozhen Xiang, Caixia Liu, Qin Lin, Pei Chen, Haipeng Song, Junhong Zhu

**Affiliations:** ^1^ School of Nursing, Shulan International Medical College, Zhejiang Shuren University, Hangzhou, People’s Republic of China.; ^2^ Department of End-of-Life Care, Zhejiang Hospital, Hangzhou, People’s Republic of China.; ^3^ Department of Nursing, Zhejiang Hospital, Hangzhou City, People’s Republic of China.; ^4^ School of Management, Zhejiang Shuren University, Hangzhou, People’s Republic of China.; ^5^ Department of Nursing Studies, Zhejiang University School of Medicine, Medical School of Zhejiang University, Hangzhou, China.

**Keywords:** breast cancer, family satisfaction, nursing competence, nursing training, palliative care

## Abstract

**Background:**

Advanced breast cancer patients often require palliative care (PC) to manage significant symptoms, relying heavily on nurses’ competence.

**Objective:**

Evaluate whether a structured PC training program can enhance nurses’ competence in breast cancer care.

**Methods:**

After an online announcement at Zhejiang Hospital, nurses enrolled in the PC training program. Due to the imbalance in trained and untrained nurses post-training, stratified randomization was applied, forming untrained (*n* = 34) and trained (*n* = 24) groups. The primary outcome, nursing competence, was assessed using Competency Inventory for Nursing Students at baseline and three months post-training. Subsequently, patients were recruited and assigned to different study groups based on the nurses providing their care. The untrained group (*n* = 167) and trained group (*n* = 106) received three months of inpatient PC care. European Organization for Research and Treatment of Cancer Quality of Life Questionnaire-C30 and Family Caregiver Satisfaction Scale-2, as secondary outcomes, were assessed at baseline and three months post-care to evaluate the training’s impact on care quality.

**Results:**

At baseline, there were no significant differences in nursing competence or patient quality of life between the trained and untrained groups. Trained nurses showed significant improvements in general clinical skills, critical thinking, and ethics compared to untrained nurses. Patients cared for by trained nurses also demonstrated improved quality of life and higher family caregiver satisfaction.

**Conclusion:**

Structured training improves nursing competence, patient quality of life, and family caregiver satisfaction.

## Introduction

Palliative care (PC) is a crucial nursing model in modern health care systems, aimed at alleviating symptoms and psychological distress in patients with severe illnesses, enhancing their families’ quality of life, and optimizing nursing practices.^1,2^ However, the implementation of PC varies across health care systems due to differences in medical resources, insurance policies, and cultural factors.^3,4^

In China, the nursing model for breast cancer patients is predominantly hospital-centered. Bone metastasis pain, malignant pleural effusion, and chemotherapy complications often lead to prolonged hospitalization. This model is driven not only by the need for symptom management but also by the health care system, insurance policies, and patient care-seeking behaviors.^5^ As tertiary hospitals dominate cancer treatment and outpatient PC services remain limited, patients are more inclined to seek continuous care through hospitalization.^6^ Additionally, inpatient treatment receives higher reimbursement rates and provides better care, making it a financially viable option. Moreover, with a high proportion of rural patients relying on large hospitals for treatment, long-term hospitalization becomes a preferred choice to reduce the inconvenience of frequent long-distance travel.^4^ Cultural beliefs reinforce this trend, as many families perceive hospitalization as offering more reliable medical support.^7^ Consequently, home-based cancer care is uncommon, making inpatient PC the primary approach for advanced breast cancer patients in China.

Despite progress in major urban centers and specialized cancer hospitals, inpatient oncology PC in China remains underdeveloped, with broader health care settings still requiring significant improvements.^8–10^ Currently, most nurses receive PC education through specialized programs, often supported by international collaborations, postgraduate studies, or workshops. Despite growing recognition of its importance, many oncology nurses still struggle to provide comprehensive PC for breast cancer patients,^11^ highlighting the urgent need for standardized training and improved care delivery. Structured training can bridge this gap by equipping nurses with the skills to better address patients’ needs.^12^ Trained nurses frequently interact with hospitalized patients, managing symptoms, pain, and emotional distress.^13^

This study utilizes the Competency Inventory for Nursing Students (CINS) to assess the primary outcome, while the European Organization for Research and Treatment of Cancer Quality of Life Questionnaire-C30 (EORTC QLQ-C30) and the Family Caregiver Satisfaction Scale (FAMCARE-2) are used to evaluate secondary outcomes.^14–16^ The study aims to provide practical evidence for PC education in breast cancer nursing in China and offer empirical support for improving patient outcomes and enhancing care quality.

## Methods

### Study overview

This Quasi-experimental study was conducted in the inpatient oncology ward of Zhejiang Hospital, a large tertiary hospital with extensive nursing resources, experienced oncology nurses, and well-equipped inpatient facilities capable of managing a high volume of cancer patients. The study was approved by the Ethics Committee of Zhejiang Shuren University (Approval No: E132-JK2024032401E35B), with all patients providing written informed consent before enrollment.

Structured PC training was conducted in Zhejiang Hospital from February to May 2023, and nurses completed the CINS assessment before and after the training. Ethical approval was obtained in May 2023, and the study commenced. Patient recruitment occurred from May 2023 to March 2024, followed by a three-month PC care, with the final cohort of patients completing care by June 2024. EORTC QLQ-C30 and FAMCARE-2 data were collected from August 2023 to June 2024, covering baseline and endpoint assessments.

### Nursing participants

All nurses were from Zhejiang Hospital and enrolled in the training program after an online announcement was posted within the hospital. After training, due to an imbalance in the number of trained and untrained nurses in the hospital, stratified randomization was applied to ensure balance in key characteristics such as gender, age, and clinical experience. The final allocation included 24 trained nurses and 34 untrained nurses, both exceeding the minimum sample size requirement of 6 per group. Eligibility criteria included holding a valid nursing qualification, at least two years of clinical experience, proficiency in breast cancer care, a stable psychological state, and the ability to work under high-pressure conditions. Exclusion criteria included nurses on extended leave, those planning department transfers, or those unable to participate due to physical or psychological conditions.

### Nurse structured training

The structured training program integrated theoretical education and practical skills training, delivered in biweekly two-hour sessions over three months. The curriculum covered breast cancer fundamentals, PC principles, pain and symptom management, side effects, communication, and psychological support. Based on NCCN Palliative Care Guidelines and Patricia Benner’s “From Novice to Expert” model, the program ensured scientific rigor and alignment with international nursing standards.^17,18^ Training methods included expert lectures (by oncologists, PC specialists, senior nursing educators, and psychologists), online resources, practical exercises, simulations, case studies, and role-playing to bridge theory and practice.

### Patient recruitment and grouping

A total of 401 patients with advanced breast cancer were recruited from 20 hospitals in Hangzhou. Inclusion criteria included a confirmed diagnosis of advanced breast cancer, a high symptom burden requiring PC, and partial self-care ability. After evaluation by oncologists at Zhejiang Hospital, 283 patients met the criteria and were admitted to the inpatient ward for PC care. Patients were assigned to different study groups based on the nurses providing their care. During the study, 10 patients withdrew for personal or family reasons, leaving 273 for analysis, with at least 13 per group, meeting the minimum sample size requirement.

### Data collection

The primary outcome of this study was the CINS scale score, which assessed nurses’ competence in ethics and accountability, general clinical skills, lifelong learning, clinical biomedical knowledge, caring, and critical thinking and reasoning. Assessments were conducted at baseline (pre-training) and three months post-training to evaluate the impact of structured PC training.

Secondary outcomes included patient quality of life assessed by the EORTC QLQ-C30 scale and family caregiver satisfaction measured by the FAMCARE-2 scale. These were evaluated at baseline (before receiving care) and three months’ post-care, ensuring a comprehensive assessment of the training’s clinical impact.

### Statistical analysis

Based on the type of variable analyzed, chi-square tests and one-sample *t*-tests were used for statistical analysis. The chi-square test was applied to categorical variables to ensure baseline balance between groups. The one-sample *t*-test was used for continuous variables, including scores from the three questionnaires, primarily to compare mean differences between groups three months post-training. The statistical hypothesis included the null hypothesis (H_0_), assuming no significant differences between groups or across time points, and the alternative hypothesis (H_1_), indicating significant differences. If the *p* value from the *t*-test was less than 0.05, the null hypothesis was rejected, confirming a significant impact of training between groups or over time.

## Results

### Baseline characteristics and group comparability

Untrained (*n* = 34) and trained (*n* = 24) nurses showed no significant differences in age, work experience, gender, ethnicity, education, certification, or professional titles (*p* > 0.05) (Table 1), and the CINS scale indicated no baseline competency differences (Supplementary Table S1).

**Table 1.** Baseline Characteristics of Nurses in the Untrained and Trained Groups

	Trained (*n* = 24)	Untrained (*n* = 34)	Comparison
Cared-for patient	4.4 ± 1.21	4.9 ± 1.03	Student’s *t-test p* = 0.099
Age	37.9 ± 4.79	36.5 ± 5.12	Student’s *t-test p* = 0.282
Years of experience	12.9 ± 5.13	11.7 ± 5.35	Student’s *t-test p* = 0.381
Gender (female, male)	21,3	26,8	χ^2^ *p* = 0.0
Race (Asian)	24	34	χ^2^ *p* = 0.189
Education (Junior college, specialized secondary schools, undergraduates)	12,8,4	15,13,6	χ^2^ *p* = 0.086
Certificate of nursing practice (Yes)	24	34	χ^2^ *p* = 0.189
Professional title (assistant, middle title, junior assistant)	10,8,6	13,7,14	χ^2^ *p* = 0.356

Data are presented as mean ± SD or counts (*n*). Student’s *t-test* was used for continuous variables, and chi-square tests were used for categorical variables. *p* < 0.05 indicates statistical significance.

SD, standard deviation.

**FIG. 1.**
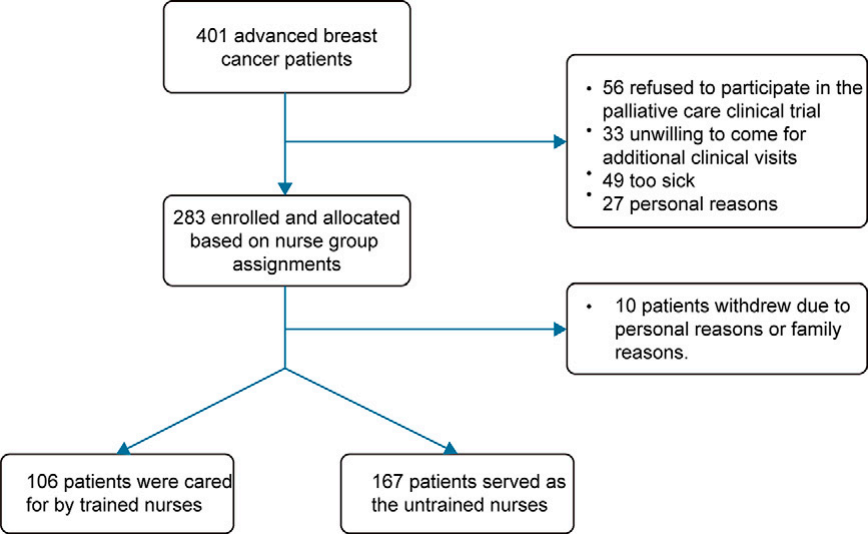
The recruitment and allocation process of breast cancer patients.

Patient recruitment is shown in Figure 1. Patients cared for by untrained (*n* = 167) and trained (*n* = 106) nurses had no significant differences in age, gender, ethnicity, or education (*p* > 0.05), and their baseline quality of life before care was also comparable (Table 2, Supplementary Table S2), confirming baseline balance.

**Table 2.** Baseline Characteristics of Patients in the Untrained and Trained Groups

	Trained (*n* = 106)	Untrained (*n* = 167)	Comparison
Age	49.4 ± 9.85	50.4 ± 9.89	*p* = 0.394 (Student’s *t*-test)
Tumor type (Breast)	106	167	χ^2^ *p* = 1.0
Stage (IV)	106	167	χ^2^ *p* = 1.0
Gender (Female, Male)	105,1	165,2	χ^2^ *p* = 1.0
Race (Asian)	106	167	χ^2^ *p* = 1.0
Education (Junior college, specialized secondary schools, junior high school, high school, primary school, undergraduates)	32,21,21,16,11,5	51,33,35,24,16,8	χ^2^ *p* = 1.0
Depression (No, Yes)	77,29	128,39	χ^2^ *p* = 0.547
Anxiety (No, Yes)	84,22	139,28	χ^2^ *p* = 0.503
Sleep disorders (Yes, No)	78,28	117,50	χ^2^ *p* = 0.624

Data are presented as mean ± SD or counts (*n*). Student’s *t-test* was used for continuous variables, and chi-square tests were used for categorical variables. *p* < 0.05 indicates statistical significance.

### Structured training improved nurses’ competence in PC delivery

**FIG. 2.**
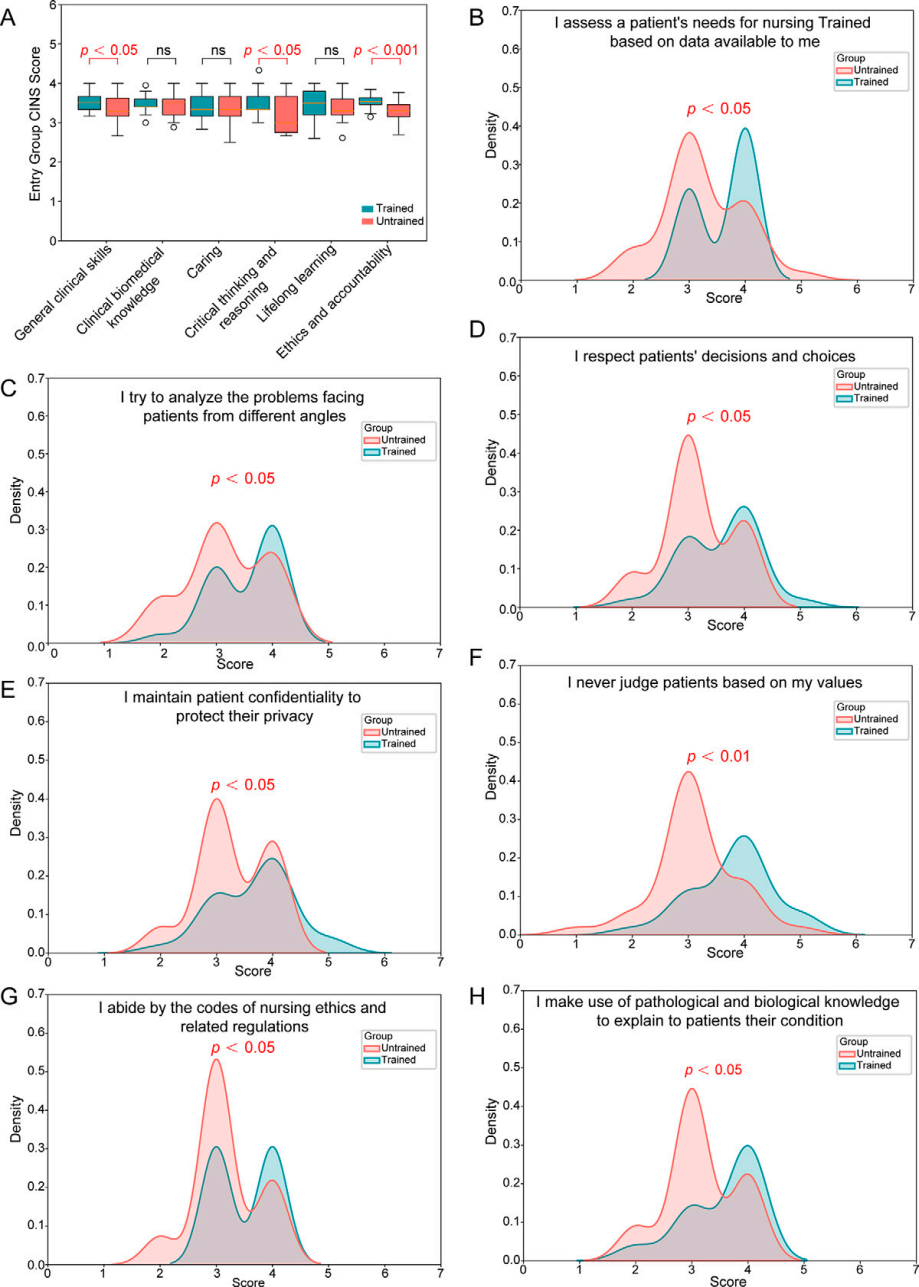
Nursing CINS scale assessment results. **(A)** Group comparisons across six nursing competency dimensions. **(B–H)** Statistical plots of CINS items with significant differences. CINS, Competency Inventory for Nursing Students.

Compared to untrained nurses, those who received structured training showed significant improvements in clinical skills, critical thinking, and ethics (Fig. 2A), including assessing patient needs (Fig. 2B), analyzing issues from multiple perspectives (Fig. 2C), and respecting patient decisions, maintaining confidentiality, and adhering to ethics (Fig. 2D–G). They also improved in applying pathological and biological knowledge to explain conditions (Fig. 2H). Details are provided in Supplementary Table S3.

### Enhanced patient-reported health and well-being

**FIG. 3.**
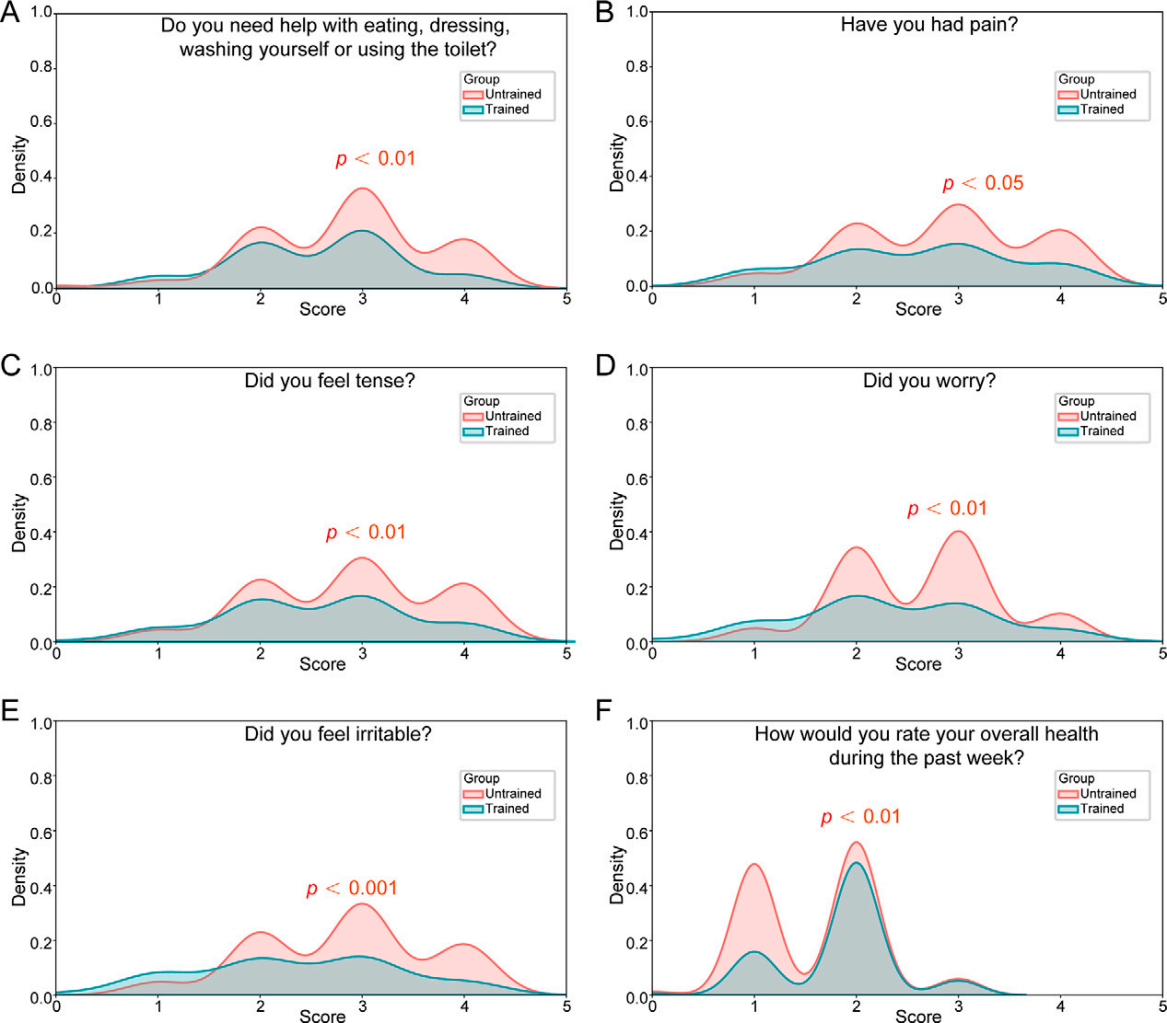
Patient-reported outcomes. **(A–F)** are statistical plots of significantly different items from the EORTC QLQ-C30 scale. EORTC QLQ, European Organization for Research and Treatment of Cancer Quality of Life Questionnaire.

The EORTC QLQ-C30 assessment results indicated that patients cared for by trained nurses showed significant improvement in daily self-care functions, including eating, dressing, washing, and toileting (*p* < 0.01) (Fig. 3A), and a notable reduction in perceived pain levels (*p* < 0.05) (Fig. 3B). In terms of emotional well-being, patients experienced significantly lower levels of tension, decreased worry, and a considerable reduction in irritability (*p* < 0.001) (Fig. 3C–E). These improvements in both health and emotional dimensions were also reflected in patients’ overall self-assessment of their health, with those under the care of trained nurses rating their health status significantly higher over the past week (*p* < 0.01) (Fig. 3F).

### Improvement in family caregiver satisfaction

**FIG. 4.**
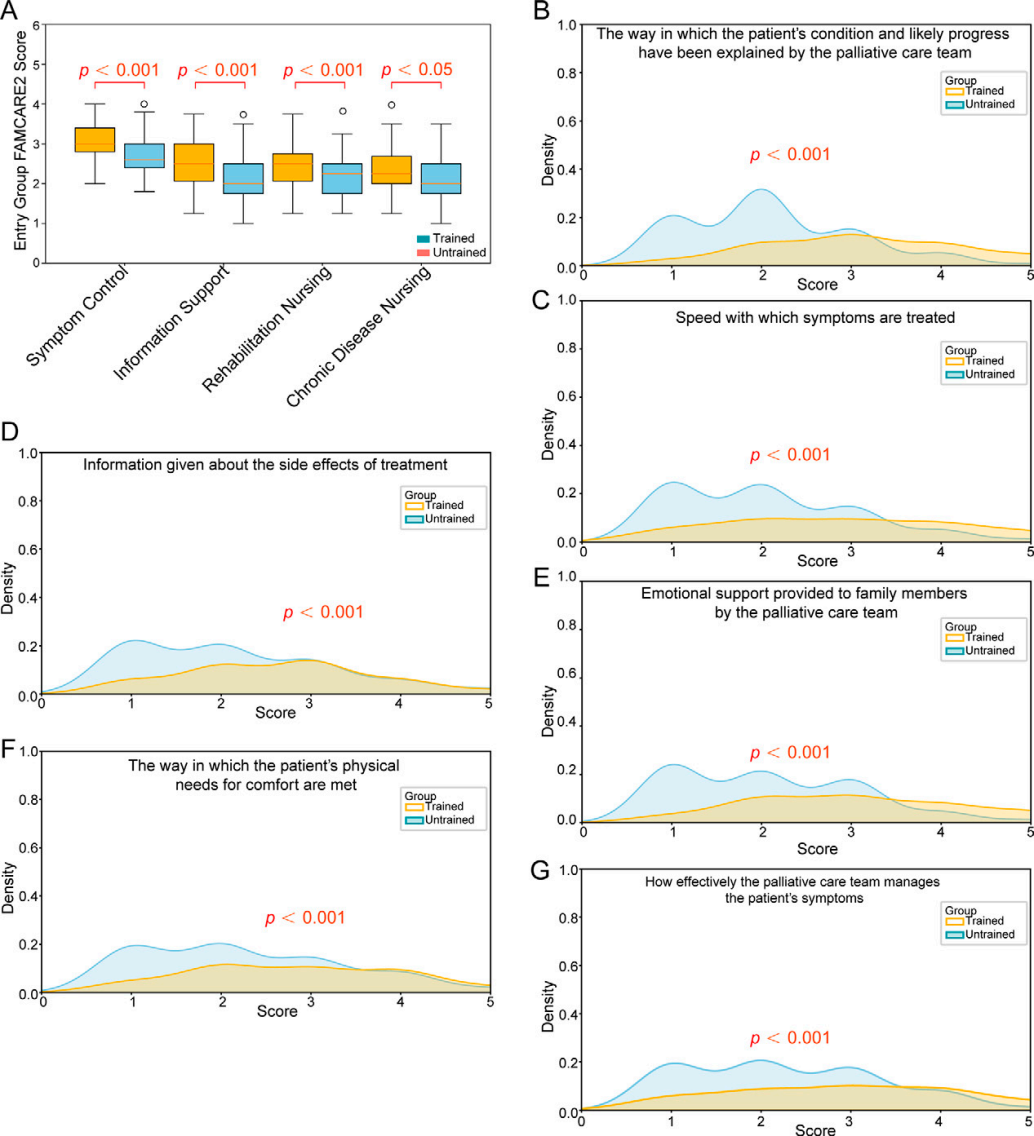
Family-reported satisfaction outcomes. **(A)** Group comparisons across four domains of the FAMCARE-2 scale. **(B–G)** Statistical plots of significantly different items from the FAMCARE-2 scale. FAMCARE-2, Family Caregiver Satisfaction Scale-2.

FAMCARE-2 scale results indicate that family caregivers of patients cared for by trained nurses reported significantly higher satisfaction across multiple key aspects, including symptom control, informational support, and rehabilitation nursing (*p* < 0.001), as well as chronic disease nursing (*p* < 0.05）(Fig. 4A). Specifically, trained nurses excelled in explaining patient conditions and prognoses, addressing symptoms promptly, providing information on treatment side effects, offering emotional support to family members, ensuring patient comfort, and enhancing overall symptom management effectiveness (*p* < 0.001) (Figure 4B–G).

## Discussion

PC is crucial in the care of hospitalized patients with advanced breast cancer, with its effectiveness highly dependent on the professional competence and ethical standards of caregivers.^19,20^ Our study aims to evaluate the impact of structured PC training on enhancing nurses’ caregiving abilities to better meet the needs of these patients.

Experimentally, nurses who underwent structured training demonstrated marked advantages in clinical skills, critical thinking, reasoning ability, and ethical responsibility. These improvements translated into higher-quality care delivery, including more effective pain management, stronger emotional support, and the promotion of patients’ self-care capabilities. Of note, these findings align with the results of Bakitas et al.^21^ and Greer JA et al.,^22^ reaffirming that systematic training yields more than just improved practices; it significantly enhances patient outcomes as well. While this study focused on improving care quality for patients, it also evaluated the broader impact of training on caregivers’ professional development and their interactions with patients.^23^ Unlike the majority of existing literature, which primarily explores patient health metrics, this research spatially expands the perspective to emphasize the dual value of structured training in enhancing both nurses’ competencies and patients’ overall quality of life.^24,25^ For instance, by establishing a trust-based relationship with caregivers, patients become more inclined to follow care recommendations, thereby yielding better outcomes.^26^

Due to cultural differences, health care system characteristics, and the need for continuous pain management, nutritional support, and surgery/radiotherapy in breast cancer patients, we adopted an inpatient care model to provide comprehensive PC. However, this study has certain limitations. Focusing on patients with advanced breast cancer allows for a deeper understanding of their care needs, but it may not fully represent the characteristics of other cancer populations. Unlike previous studies that primarily focused on patient health metrics, this study emphasizes the dual impact of structured training in enhancing both nurses’ competencies and patients’ overall quality of life.^27–29,24,25^ Additionally, the imbalance in the number of trained and untrained nurses may introduce selection bias and workload differences. To mitigate these effects, we applied stratified randomization to balance key baseline characteristics and ensured an adequate sample size. Furthermore, the patient-to-nurse ratio remained within clinical standards, minimizing the likelihood that differences in-patient outcomes were solely due to workload variations.

Overall, structured PC training enhances nurses’ professional skills, improves patient care, and enhances quality of life.^30^ Future efforts should focus on optimizing training content and adapting it to various diseases and cultural contexts.

## Ethical Statement

This study received approval from the Ethics Committee of Medical and Life Sciences at Zhejiang Shuren University (Approval No. E132-JK2024032401E35B).

## Abbreviations Used

CINS: Competency Inventory for Nursing Students
EORTC QLQ-C30: European Organization for Research and Treatment of Cancer Quality of Life Questionnaire C30
FAMCARE-2: Family Caregiver Satisfaction Scale
PC: Palliative Care
