# Supplementary material for: Structured Palliative Care Training Enhances Nursing Competence: Evidence from Breast Cancer Care
Source: Palliat Med Rep. 2025 Apr 29;6(1):196–204. doi: 10.1089/pmr.2024.0061 (PMC12411899; doi:10.1089/pmr.2024.0061)
Supplement: Supplementary Table S2 [file pmr.2024.0061_supplementarytables2.docx]

**Table S2 Baseline quality of life levels of patients before receiving care from trained and untrained nurses** **in the trained and untrained groups**

| **Entry** | **trained group (n = 106)** | **untrained group (n = 167)** | **Comparison** |
| --- | --- | --- | --- |
| Do you have any trouble doing strenuous activities, like carrying a heavy shopping bag or a suitcase? | 2.8±1.18 | 2.7±0.89 | P=0.68 (NS by Student's t-test) |
| Do you have any trouble taking a long walk? | 2.6±1.17 | 2.5±0.88 | P=0.79 (NS by Student's t-test) |
| Do you have any trouble taking a short walk outside of the house? | 2.8±1.07 | 2.8±0.82 | P=0.61 (NS by Student's t-test) |
| Do you need to stay in bed or a chair during the day? | 2.8±1.26 | 2.7±0.84 | P=0.39 (NS by Student's t-test) |
| Do you need help with eating, dressing, washing yourself or using the toilet? | 2.9±1.27 | 2.8±0.85 | P=0.45 (NS by Student's t-test) |
| Were you limited in doing either your work or other daily activities? | 2.8±1.21 | 2.7±0.94 | P=0.42 (NS by Student's t-test) |
| Were you limited in pursuing your hobbies or other leisure time activities? | 2.9±1.18 | 2.8±0.9 | P=0.38 (NS by Student's t-test) |
| Were you short of breath? | 2.8±1.15 | 2.8±0.86 | P=0.63 (NS by Student's t-test) |
| Have you had pain? | 2.8±1.21 | 2.9±0.88 | P=0.64 (NS by Student's t-test) |
| Did you need to rest? | 2.5±1.22 | 2.6±0.97 | P=0.96 (NS by Student's t-test) |
| Have you had trouble sleeping? | 2.8±1.2 | 2.8±0.88 | P=0.68 (NS by Student's t-test) |
| Have you felt weak? | 2.8±1.14 | 2.9±0.81 | P=0.51 (NS by Student's t-test) |
| Have you lacked appetite? | 2.9±1.16 | 2.8±0.82 | P=0.66 (NS by Student's t-test) |
| Have you felt nauseated? | 2.6±1.17 | 2.6±0.94 | P=0.84 (NS by Student's t-test) |
| Have you vomited? | 2.8±1.2 | 2.8±0.87 | P=0.92 (NS by Student's t-test) |
| Have you been constipated? | 2.7±1.24 | 2.8±0.86 | P=0.57 (NS by Student's t-test) |
| Have you had diarrhea? | 2.6±1.12 | 2.7±0.94 | P=0.63 (NS by Student's t-test) |
| Were you tired? | 2.7±1.22 | 2.8±0.86 | P=0.84 (NS by Student's t-test) |
| Did pain interfere with your daily activities? | 2.9±1.24 | 2.9±0.88 | P=0.72 (NS by Student's t-test) |
| Have you had difficulty in concentrating on things, like reading a newspaper or watching television? | 2.7±1.18 | 2.7±0.93 | P=0.96 (NS by Student's t-test) |
| Did you feel tense? | 2.8±1.12 | 2.9±0.87 | P=0.7 (NS by Student's t-test) |
| Did you worry? | 2.6±1.23 | 2.6±0.76 | P=0.63 (NS by Student's t-test) |
| Did you feel irritable? | 2.8±1.19 | 2.8±0.86 | P=0.63 (NS by Student's t-test) |
| Did you feel depressed? | 2.8±1.22 | 2.7±0.9 | P=0.84 (NS by Student's t-test) |
| Have you had difficulty remembering things? | 2.8±1.17 | 2.8±0.91 | P=0.57 (NS by Student's t-test) |
| Has your physical condition or medical treatment interfered with your family life? | 2.7±1.17 | 2.7±0.91 | P=0.72 (NS by Student's t-test) |
| Has your physical condition or medical treatment interfered with your social activities? | 2.9±1.27 | 2.8±0.9 | P=0.77 (NS by Student's t-test) |
| Has your physical condition or medical treatment caused you financial difficulties? | 2.7±1.18 | 2.7±0.91 | P=0.72 (NS by Student's t-test) |
| How would you rate your overall health during the past week? (1-7, 1 being very poor and 7 being excellent) | 1.7±1.08 | 1.6±0.61 | P=0.49 (NS by Student's t-test) |
| How would you rate your overall quality of life during the past week? (1-7, 1 being very poor and 7 being excellent) | 1.9±1.05 | 1.7±0.49 | P=0.06 (NS by Student's t-test) |

Notes: The baseline quality of life of patients was assessed using the EORTC QLQ-C30 questionnaire, and there was no significant difference in the quality of life between the control group and the intervention group at baseline. Data are presented as mean ± SD or counts (n). Student’s t-test was used for continuous variables, and chi-square tests were used for categorical variables. p < 0.05 indicates statistical significance.
