# Supplementary material for: Structured Palliative Care Training Enhances Nursing Competence: Evidence from Breast Cancer Care
Source: Palliat Med Rep. 2025 Apr 29;6(1):196–204. doi: 10.1089/pmr.2024.0061 (PMC12411899; doi:10.1089/pmr.2024.0061)
Supplement: Supplementary Table S3 [file pmr.2024.0061_supplementarytables3.docx]

**Table S3 Comparison of CINS scores for untrained and trained group nurses after three months of training**

| **Entry** | **trained group (n = 24)** | **untrained group(n=34)** | **Comparison** |
| --- | --- | --- | --- |
| I make use of pathological and biological knowledge to explain to patients their condition. | 3.5±0.66 | 3.2±0.63 | *p* = 0.04 |
| I explain to patients the procedure and results of physical examinations. | 3.6±0.65 | 3.4±0.7 | *p* = 0.44 (NS by Student's t-test) |
| I understand the mechanism, side effects, and clinical applications of the medicine patients are taking. | 3.4±0.77 | 3.3±0.64 | *p* = 0.78 (NS by Student's t-test) |
| I understand the purposes and normal values of clinical examination. | 3.4±0.5 | 3.4±0.6 | *p* = 0.82 (NS by Student's t-test) |
| I understand the principles and logic of medical treatment. | 3.5±0.51 | 3.5±0.66 | *p* = 0.66 (NS by Student's t-test) |
| I collect as much information about the patient as possible to formulate the best nursing strategy. | 3.6±0.58 | 3.3±0.68 | *p* = 0.14 (NS by Student's t-test) |
| I assess a patient's needs for nursing intervention based on data available to me. | 3.6±0.49 | 3.2±0.7 | *p* = 0.02 |
| I provide patients with nursing treatments that suit their needs. | 3.7±0.48 | 3.4±0.6 | *p* = 0.06 (NS by Student's t-test) |
| I assess the outcome of my nursing interventions. | 3.4±0.58 | 3.3±0.67 | *p* = 0.37 (NS by Student's t-test) |
| I set priorities of nursing tasks based on the needs of patients. | 3.5±0.59 | 3.4±0.56 | *p =* 0.91 (NS by Student's t-test) |
| I try to provide comprehensive follow-up medical care that suits the needs of patients. | 3.4±0.65 | 3.4±0.6 | *p* = 0.84 (NS by Student's t-test) |
| I carefully consider the condition of patients and make reasonable judgments and inferences. | 3.4±0.77 | 3.2±0.78 | *p* = 0.5 (NS by Student's t-test) |
| I try to look for the root cause of any changes in patient's condition. | 3.5±0.59 | 3.2±0.54 | *p* = 0.05 (NS by Student's t-test) |
| I try to analyze the problems facing patients from different angles. | 3.5±0.59 | 3.2±0.72 | *p* = 0.04 |
| I try to empathize with patients in order to identify their needs for medical care. | 3.5±0.51 | 3.4±0.7 | *p* = 0.78 (NS by Student's t-test) |
| I encourage patients to express their feelings and I am attentive to their positive and negative feelings. | 3.3±0.55 | 3.3±0.63 | *p* = 0.99 (NS by Student's t-test) |
| I provide timely emotional support for patients when necessary. | 3.4±0.65 | 3.5±0.79 | *p* = 0.78 (NS by Student's t-test) |
| I pay attention to the psychological, social, and spiritual wellbeing of patients. | 3.5±0.66 | 3.4±0.78 | *p* = 0.81 (NS by Student's t-test) |
| I try my best to provide patients with a comfortable and peaceful environment. | 3.4±0.5 | 3.5±0.66 | *p* = 0.74 (NS by Student's t-test) |
| I carry out doctor's instructions and nursing care routine accurately. | 3.3±0.64 | 3.5±0.75 | *p* = 0.47 (NS by Student's t-test) |
| I believe that each patient's life has its own meaning. | 3.4±0.71 | 3.3±0.64 | *p* = 0.77 (NS by Student's t-test) |
| I actively fulfill my duties. | 3.4±0.58 | 3.3±0.68 | *p* = 0.64 (NS by Student's t-test) |
| I perform nursing tasks as much as possible to avoid patient injury. | 3.4±0.72 | 3.2±0.7 | *p* = 0.34 (NS by Student's t-test) |
| Before carrying out any nursing interventions, I consider the rights and interests of patients. | 3.5±0.51 | 3.4±0.6 | *p* = 0.62 (NS by Student's t-test) |
| I respect patients' decisions and choices. | 3.6±0.65 | 3.2±0.63 | *P* = 0.02 |
| I maintain patient confidentiality to protect their privacy. | 3.7±0.7 | 3.3±0.63 | *P* = 0.04 |
| I never judge patients based on my values. | 3.8±0.72 | 3.1±0.73 | *P* = 0.0 |
| I respect patients' beliefs and values. | 3.5±0.66 | 3.4±0.74 | *p* = 0.5 (NS by Student's t-test) |
| I abide by the codes of nursing ethics and related regulations. | 3.5±0.51 | 3.2±0.58 | *p* = 0.03 |
| I am accountable for my professional judgments and actions. | 3.4±0.5 | 3.3±0.58 | *p* = 0.41 (NS by Student's t-test) |
| I am fully aware of the limitations of my professional role and abilities. | 3.5±0.66 | 3.2±0.58 | *p* = 0.05 (NS by Student's t-test) |
| I work meticulously and conscientiously. | 3.7±0.48 | 3.4±0.61 | *p* = 0.14 (NS by Student's t-test) |
| I make effective use of my time at work. | 3.5±0.51 | 3.4±0.65 | *p* = 0.51 (NS by Student's t-test) |
| I am fully aware of what I need to learn. | 3.5±0.72 | 3.2±0.64 | *p* = 0.11 (NS by Student's t-test) |
| I enjoy seeking answers to questions. | 3.5±0.66 | 3.4±0.85 | *p* = 0.48 (NS by Student's t-test) |
| I set goals for my learning. | 3.6±0.49 | 3.5±0.66 | *p* = 0.44 (NS by Student's t-test) |
| I know where and how to look for resources for learning. | 3.4±0.78 | 3.5±0.79 | *p* = 0.8 (NS by Student's t-test) |
| I make use of technology and other resources in learning. | 3.3±0.69 | 3.3±0.64 | *p* = 0.86 (NS by Student's t-test) |

Notes: Data are presented as mean ± SD or counts (n). Student’s t-test was used for continuous variables, and chi-square tests were used for categorical variables. *p* < 0.05 indicates statistical significance.
